# Valproic Acid Promotes the Differentiation of Satellite Glial Cells into Neurons via the pH-Dependent Pathway

**DOI:** 10.3390/biom15070986

**Published:** 2025-07-11

**Authors:** Dongyan Wang, Wenrun Kang, Jinhui Zhang, Jianwei Xu, Ruyi Wang, Xiangdan Xiao, Chao Wei, Wenfeng Yu, Junhou Lu

**Affiliations:** 1 Department of Physiology, School of Basic Medicine, Guizhou Medical University, Guiyang 550025, China; wdyan@gmc.edu.cn (D.W.); 18216663082@163.com (W.K.); v3uqjgqcxq@163.com (R.W.); 17785172159@163.com (X.X.); 15685414115@163.com (C.W.); 2 Department of Pharmacology, School of Basic Medicine, Guizhou Medical University, Guiyang 550025, China; 15628006853@163.com (J.Z.); xujianwei@gmc.edu.cn (J.X.); 3 Center for Tissue Engineering and Stem Cell Research, Guizhou Medical University, Guiyang 550025, China; 4 Key Laboratory of Molecular Biology, Guizhou Medical University, Guiyang 550001, China; 5 Key Laboratory of Human Brain Bank for Functions and Diseases, Department of Education of Guizhou Province, Guizhou Medical University, Guiyang 550025, China

**Keywords:** VPA, satellite glial cells, neuronal differentiation, intracellular pH, NHE1

## Abstract

Valproic acid (VPA) is a widely prescribed antiepileptic agent whose teratogenic potential has been recognized. In recent years, VPA has been shown to promote neuronal regeneration; however, the exact molecular mechanisms are not fully understood. This study elucidates the pH-dependent pathway through which VPA promotes the differentiation of satellite glial cells (SGCs) into neurons. We observed sustained intracellular pH elevation during the VPA-induced neural differentiation of SGCs, and the modulation of intracellular pH was shown to influence this differentiation process. Then, we found that VPA regulates intracellular pH through NHE1 (sodium–hydrogen exchanger 1), and that the pharmacological inhibition of NHE1 not only attenuated intracellular pH elevation but also substantially impaired VPA-induced neuronal differentiation. Finally, our results showed that the elevated intracellular pH promoted the neuronal differentiation of SGCs by activating β-catenin signaling. These findings provide novel insights into the mechanisms of VPA-induced neurogenesis, advancing our understanding of its pharmacological profile and informing its potential therapeutic application in neuronal regeneration strategies.

## 1. Introduction

Valproic acid (VPA) is a short-chain fatty acid and an antiepileptic drug that is commonly used in clinical practice [1,2]. Many studies have shown that VPA contributes to neuronal regeneration. For example, VPA can induce the neuronal differentiation of hippocampal and cortical neural stem cells by regulating the histone acetylation of neuron-specific transcription factors (such as *Ngn1*) [3,4]. VPA can effectively transdifferentiate astrocytes and fibroblasts into neurons by combining with Repsox (TGF-β receptor inhibitor) [5], CHIR99021 (GSK-3 inhibitor), SP600125 (JNK inhibitor), GO6983 (PKC inhibitor), Forskolin (Adenylate cyclase activator), and Y-27632 (ROCK inhibitor) [6]. Our group also found that VPA combined with CHIR99021, RO4929097 (γ secretase inhibitor), and SU5402 (multi-targeted receptor tyrosine kinase inhibitor) can promote the transformation of satellite glial cells (SGCs) into sensory neurons, and that VPA plays a crucial role in this process [7]. However, the specific mechanism by which VPA promotes neuronal regeneration is still not fully understood.

In normal mammalian cells, the intracellular pH has long been considered stable, usually being maintained between 7.2 and 7.4. Only in disease states will the intracellular pH be dysregulated; for example, in cancer cells, the pH value will increase. However, in recent years, new evidence has shown that the intracellular pH of normal cells also changes dynamically, and that this change plays an important role in the differentiation of normal mammalian cells [8,9]. Studies have found that a decrease in the intracellular pH hinders the differentiation of Drosophila follicle stem cells; on the contrary, an increase in the intracellular pH has been shown to promote the differentiation of mouse embryonic stem cells (mESCs) [10]. Therefore, an increased intracellular pH is also considered to be a key condition for the differentiation of adult epithelial cells and embryonic stem cells.

It has been reported that as the intracellular pH decreases, histones become globally hypoacetylated in an HDAC (histone deacetylase)-dependent manner; whereas as the intracellular pH increases, histones become globally hyperacetylated [11]. Acetylation modification is generally considered to be associated with transcriptional activation, which means that the intracellular pH may regulate the activation of gene transcription by affecting the acetylation state of proteins, thereby participating in the regulatory process of cell differentiation. As a histone deacetylase inhibitor, it is not clear whether VPA affects the intracellular pH and whether it exerts its effects via the regulation of the intracellular pH.

In this study, we found that VPA can increase the intracellular pH of SGCs and human glial cell line SVGp12 cells by using intracellular pH indicators, and that this change is involved in the VPA-induced neuronal differentiation process. Moreover, our results also revealed that VPA affects the intracellular pH by regulating NHE1 (sodium–hydrogen exchanger 1), and that changes in the intracellular pH ultimately regulate the activation of β-catenin signaling. This study elucidates the mechanism via which VPA promotes neuronal differentiation from the perspective of the intracellular pH, enhances our understanding of the mechanism via which VPA regulates neuronal regeneration, and provides a new theoretical basis for its clinical application.

## 2. Materials and Methods

### 2.1. DRG (Dorsal Root Ganglia)-SGCs Culture

DRG-SGCs were cultured as described previously [7]. Briefly, DRGs were isolated from Sprague Dawley (SD) rats (post-natal 1–2 d). Then, the DRGs were plated at a density of approximately 30 explants per 60 mm dish in DRG-SGCs medium. When a large number of cells had migrated from the DRG explants (about 10 days), the cells were passaged and seeded into the poly-L-lysine (PLL)-coated plates (Sigma, P7280–5mg, Sigma-Aldrich, St. Louis, MO, USA) at a density of 100,000 cells per well in a 35 mm dish. The culture medium was changed every 2 days. The DRG-SGC medium comprised DMEM/F12 (Corning, 10–092-CVR), 2% B27 (Invitrogen, 17504–044), L-Glutamine (Gibco, Thermo Fisher Scientific, Waltham, MA, USA), penicillin/streptomycin (Gibco), 0.3 mg/mL BSA (Sigma, A1933), 10^−6^ M Insulin (Sigma, I1882), 10 ng/mL T4 (MCE, HY-18341), 10 ng/mL T3 (Sigma, T2877), 38 ng/mL Dexamethasone (Sigma, D4902), and 20 ng/mL NRG1 (neuregulin1, 11609-H01H2, Sino Biological Inc., Beijing, China).

### 2.2. SVGp12, U87, and U251 Cell Culture

The glial cell line SVGp12 and glioma cell lines U87 and U251, purchased from Hunan Fenghui Biotechnology Co., Ltd. (Changsha, China). Cells, were cultured in Dulbecco’s modified Eagle medium (DMEM, Gibco, 12100046) supplemented with 10% fetal bovine serum (FBS, Gibco, A5256701) at 37 °C in an atmosphere of 5% CO_2_. The media was changed every 2 days, and cells were passaged at approximately 80% confluency.

### 2.3. Neuronal Induction

After cell passage for 24 h, the culture medium was replaced with neuronal differentiation medium (NDM) and refreshed every 3 days. The NDM comprised neurobasal medium/DMEM/F12 (1:1), 2% B27, 1% N2 (Gibco, 17502–048), 200 μM of ascorbic acid (Sigma, A5960–25G), and 3 mM of VPA (Sigma, P4543–10G).

### 2.4. Cytosolic pH Measurement

In order to measure the intracellular pH, the cells were incubated with BCECF AM (1 μΜ, Beyotime Biotechnology, Shanghai, China, S1006) for 30 min in a humidified incubator gassed with 5% CO_2_ according to the manufacturer’s protocol. Fluorescence images were captured via laser confocal microscopy (Olympus Corporation, Tokyo, Japan) under the same conditions, and the fluorescent intensity of each cell was analyzed using ImageJ (National Institutes of Health, Bethesda, MD, USA). Specifically, four random fields (≥30 cells/field) per group were captured. The average grayscale value of single-cell Regions of Interest (ROIs) was analyzed using the ImageJ software (version 1.53t, National Institutes of Health, Bethesda, MD, USA) across three independent experiments. All single-cell data were pooled for statistical analysis and presented as scatter plots with medians.

In addition, flow cytometry was used to obtain the fluorescence intensity of each group of cells. Specifically, SGCs were stained with BCECF-AM (1 μM, 37 °C, 30 min) and analyzed using a CytoFLEX flow cytometer (Beckman Coulter, Brea, CA, USA; 488 nm laser, FITC 525/40 nm filter), collecting ≥10,000 events per sample. Analysis Workflow: (1) Debris Removal: FSC-A/SSC-A gating to exclude debris. (2) Doublet Discrimination: FITC-A/FITC-Width gating to exclude cell aggregates. (3) Doublet Exclusion: FITC-A/FITC-H gating to remove doublets. (4) (Threshold Setting: The threshold was defined as the upper limit containing 99% of negative events based on the autofluorescence from unstained control cells of the NDM group) (B525-FITC-A = 4.7 × 10^3^ a.u.). (5) Uniform Application: This identical threshold was applied to all the samples using the “Apply to All Samples” function in the FlowJo software (version 10.8.1, BD Biosciences, Franklin Lakes, NJ, USA). (6) BCECF^+^ Population Definition: Events to the right of the threshold were defined as the high-BCECF-fluorescence population (P2). (7) Statistical Metric: The final reported metric is the percentage of P2 cells within the total single-cell population (%P2).

### 2.5. Immunofluorescence

The cultures were immunostained as described previously [7]. After being fixed with 4% paraformaldehyde, the cells were blocked with 0.2% Triton X-100 and 5% BSA. Primary antibodies (Tuj1, 1:500, Covance, BioLegend, San Diego, CA, USA, MMS435P; NHE1, 1:200, Abcam, Cambridge, UK, ab67314) were incubated with the slides at 4 °C overnight. After PBS washes, the appropriate secondary antibody (488-labeled Goat Anti-Rabbit IgG(H + L) or Cy3-labeled Goat Anti-Mouse/Rabbit IgG(H + L), 1:1500, Beyotime Biotechnology, Shanghai, China) was incubated with the slides for 2 h at room temperature. Cell nuclei were counterstained with Hoechst 33,258 (1:1000, Invitrogen, Thermo Fisher Scientific, Waltham, MA, USA). Fluorescent images were generated via laser confocal microscopy (Olympus Corporation, Tokyo, Japan) under the same conditions. The controls, which were treated with nonspecific mouse/rabbit IgM or secondary antibodies alone, showed no staining.

### 2.6. Western Blot

Western blot procedures were performed as previously described [12]. Briefly, the cells were lysed with RIPA buffer with protease and phosphatase inhibitor cocktails and then centrifuged at 12,000 rpm for 15 min to remove cell debris. After determining the protein concentration using the BCA assay kit (Applygen Technologies Inc., Beijing, China), identical quantities (20–30 µg) of protein lysates were separated in 10% gels using SDS-PAGE and transferred to polyvinylidene difluoride membranes (Millipore, Merck KGaA, Darmstadt, Germany, IPVH00010) using a Trans-Blot SD Semi-Dry Electrophoretic Transfer Cell (Bio-Rad Laboratories, Hercules, CA, USA). Subsequently, according to the molecular size of the target protein, the blots were cut prior to being hybridized with antibodies. After being blocked with 5% non-fat milk in TBST, the membranes were incubated overnight with primary antibodies at 4 °C. The primary antibodies used were mouse anti-Tuj1 (Tuj1, 1:3000, Covance, MMS435P), rabbit anti-β-catenin (1:1000, CST, 8480), rabbit anti-Non-phospho (Active) β-catenin (ABC, 1:1000, CST, 8814), and mouse anti-GAPDH (Abcam, ab8245). After being washed three times with TBST, the membranes were incubated for 1 h at room temperature with appropriate horseradish peroxidase-conjugated secondary antibodies (1:2000 dilution; Cell Signaling Technology, Danvers, MA, USA). Antigen–antibody complexes were visualized using ECL after they had been washed three times with TBST. The intensity of the immunoreactive bands was quantified using a gel image analysis software (ImageJ_1.32J, National Institutes of Health, Bethesda, MD, USA).

### 2.7. Statistical Analysis

The data were analyzed using the GraphPad software (version 9.0, GraphPad Software Inc., San Diego, CA, USA). The statistical significance of the differences was assessed using one-way analysis of variance (ANOVA), followed by Tukey’s test. The data are presented as the mean ± SD of at least 3 independent experiments. A level of *p*  <  0.05 was considered statistically significant.

## 3. Results

### 3.1. The Process of VPA-Induced Differentiation of SGCs into Neurons Is Accompanied by a Sustained Increase in the Intracellular PH

**Figure 1:**
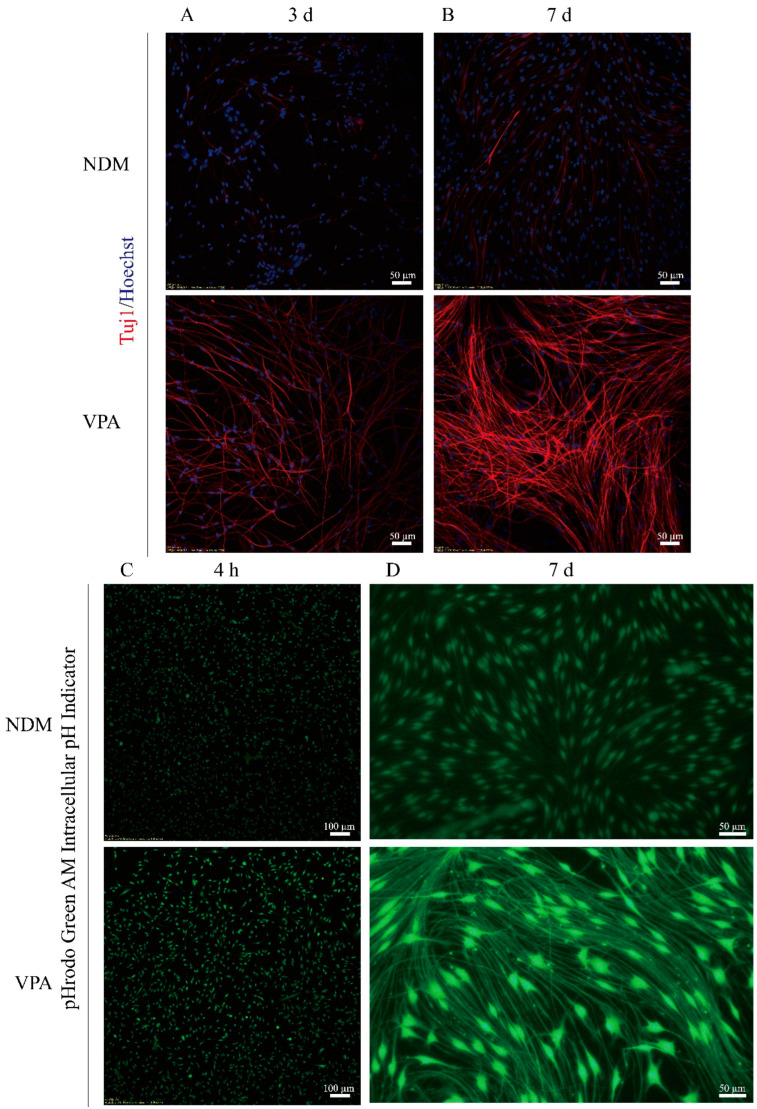
The VPA-induced differentiation of SGCs into neurons is accompanied by a sustained increase in the intracellular pH. After passage, the SGCs were divided into two groups: the NDM group (neuronal differentiation basic medium +PBS) and the VPA group (neuronal differentiation basic medium +VPA). After treatment for 3 days (**A**) and 7 days (**B**), the cells were fixed with paraformaldehyde for immunofluorescence staining. (**A**,**B**) Representative images of immunofluorescence staining at 3 days and 7 days. (**C**,**D**) Representative images after incubation with the pH indicator BCECF. Note that the brighter the fluorescence intensity, the higher the intracellular pH.

In our previous studies on the differentiation of SGCs into neurons, we observed that VPA significantly promoted the neuronal differentiation of SGCs [7]. However, the underlying molecular mechanisms remain unclear. Therefore, this study focuses on the molecular mechanism via which VPA promotes the neuronal differentiation of SGCs. First, using immunofluorescence staining, we found that compared with the NDM group, the expression of neuron-specific marker Tuj1 was significantly increased in the VPA-treated group (Figure 1A,B), indicating that VPA significantly promoted the differentiation of SGCs into neurons. Subsequently, by using the intracellular pH indicator BCECF, we found that compared with the NDM group, the fluorescence intensity of SGCs was significantly enhanced in the VPA-treated group after 4 h and 7 d of treatment (Figure 1C,D), suggesting that VPA treatment can lead to a significant increase in the intracellular pH of SGCs, as a higher BCECF fluorescence intensity corresponds to an elevated intracellular pH. These results demonstrate that the VPA-induced differentiation of SGCs into neurons is accompanied by a sustained increase in the intracellular pH.

### 3.2. Intracellular pH Elevation Mediates VPA-Induced Neuronal Differentiation

**Figure 2:**
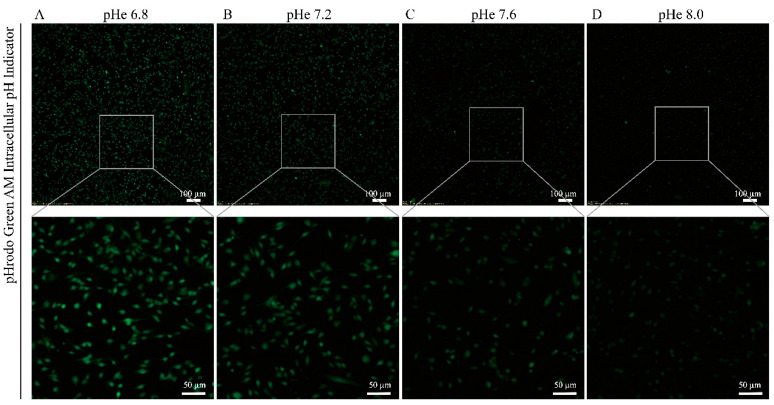
The intracellular pH in SGCs progressively decreases with elevated extracellular pH. The NDM was adjusted to four pHs: 6.8, 7.2, 7.6, and 8.0. Following 4 h of incubation, the intracellular pH was measured using pH-sensitive fluorescent indicators BCECF. (**A**–**D**) Representative images after incubation with the pH indicator BCECF.

Studies have shown that the extracellular pH (pHe) affects the intracellular pH [13,14]. To investigate this relationship, we adjusted the culture medium to four distinct pH levels (pHe 6.8, 7.2, 7.6, and 8.0) and assessed their effects on the intracellular pH. Intriguingly, higher extracellular pH levels correlated with a reduced intracellular fluorescence intensity (Figure 2), indicating a paradoxical decrease in the intracellular pH as the extracellular pH increased. This inverse relationship confirms that extracellular pH fluctuations directly influence the intracellular pH in an antagonistic manner.

**Figure 3:**
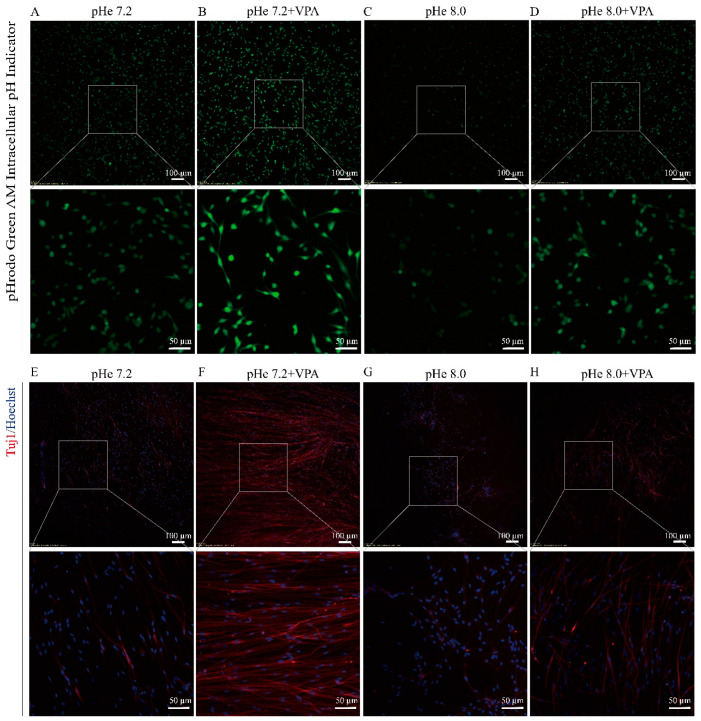
Reduced intracellular pH impairs VPA-induced neuronal differentiation: (**A**–**D**) Representative images after incubation with pH indicator BCECF. The NDM was adjusted to pH 7.2 and 8.0, followed by VPA treatment. After 4 h of incubation, the intracellular pH was assessed using the pH-sensitive fluorescent indicator BCECF. (**E**–**H**) Representative images of immunofluorescence staining after 3 days.

**Figure 4:**
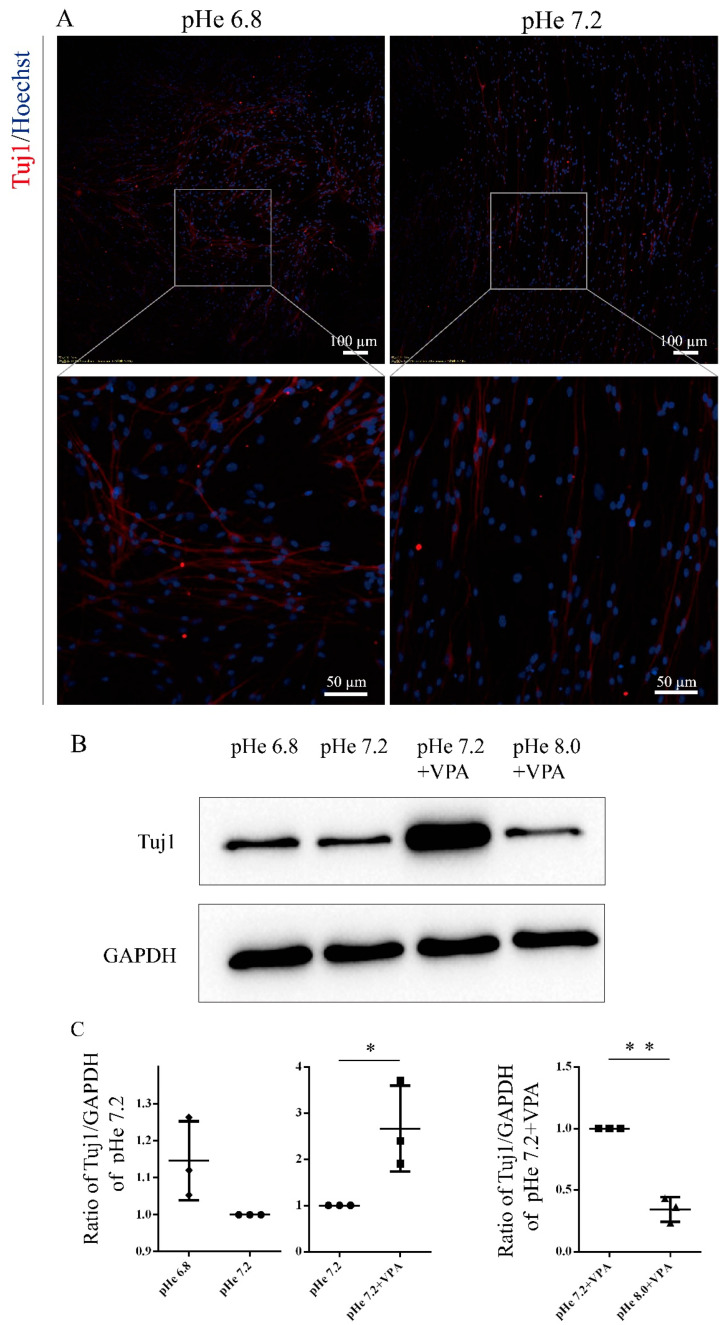
Intracellular pH mediates the VPA-induced neuronal differentiation of SGCs: (**A**) Representative images of immunofluorescence staining. The NDM was adjusted to pH 6.8 and 7.2. After incubation for 3 days, immunofluorescence staining was performed. (**B**) Representative images of Western blot. The SGCs were divided into four groups: pHe 6.8, pHe 7.2, pHe 7.2 + VPA, and pHe 8.0 + VPA. After 3 days, Western blot analysis was performed. (**C**) Statistical analysis. The ImageJ software was used to calculate the ratio of Tuj1/GAPDH, and the ratio of Tuj1/GAPDH in the control was normalized to 1. The statistical significance of the differences was assessed using one-way analysis of variance (ANOVA), followed by Tukey’s test. The data are presented as the mean ± SD of at least three independent experiments. pHe 6.8 vs. pHe 7.2: *p* = 0.9800; pHe 7.2 vs. pHe 7.2 + VPA: *p* = 0.0102, *; pHe 7.2 + VPA vs. pHe 8.0 + VPA: *p* = 0.0064, **. Original Western blot images can be found in Figure S1.

Based on the above observations, we adjusted the extracellular pH to verify whether VPA promotes the differentiation of SGCs into neurons by upregulating the intracellular pH. The confocal microscopy revealed that SGCs treated with pHe 7.2 + VPA significantly enhanced the intracellular fluorescence intensity compared to the pHe 7.2 control (Figure 3A,B), confirming that VPA elevates the intracellular pH. Conversely, when the pHe was adjusted to 8.0, the intracellular fluorescence intensity decreased sharply (Figure 3C,D), reflecting the acidification of the intracellular environment. Critically, immunofluorescence staining demonstrated that lowering the intracellular pH (via pHe 8.0) suppressed the VPA-induced upregulation of Tuj1 expression in SGCs (Figure 3E,H). In contrast, reducing the extracellular pH to 6.8 elevated the intracellular pH and enhanced the expression of Tuj1 (Figure 2 and Figure 4A). Western blot analysis further validated these findings: pHe 6.8 treatment increased the expression of Tuj1 in SGCs compared with pHe 7.2 (Figure 4B,C); meanwhile, pHe 8.0 + VPA attenuated Tuj1 levels relative to pHe 7.2 + VPA (Figure 4B,C). These results indicate that an elevated intracellular pH promotes the neuronal differentiation of SGCs, whereas a reduction in the intracellular pH impairs VPA-driven differentiation, suggesting that VPA promotes the differentiation of SGCs into neurons by increasing the intracellular pH.

### 3.3. VPA Increases Intracellular PH by Enhancing the Expression of NHE1

**Figure 5:**
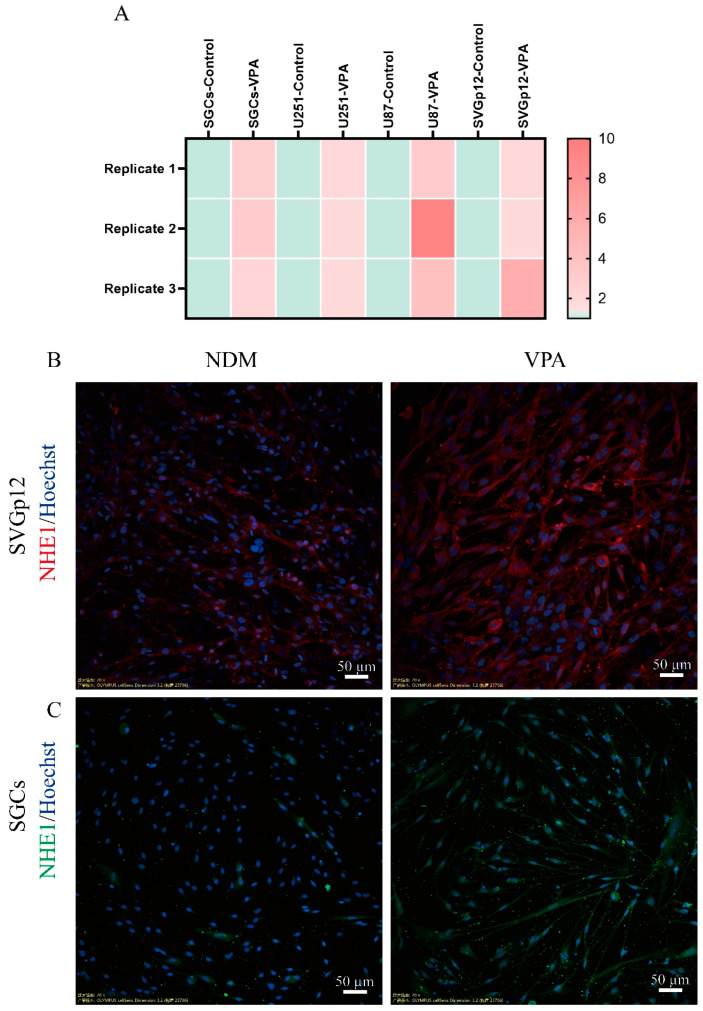
VPA upregulated the expression of NHE1: (**A**) The hotspot map shows the expression of *NHE1* in SGCs, U251, U87, and SVGp12 cells after VPA treatment for 3 days. (**B**,**C**) Representative images of immunofluorescence staining. After treatment for 3 days, the cells were fixed with paraformaldehyde for immunofluorescence staining.

**Figure 6:**
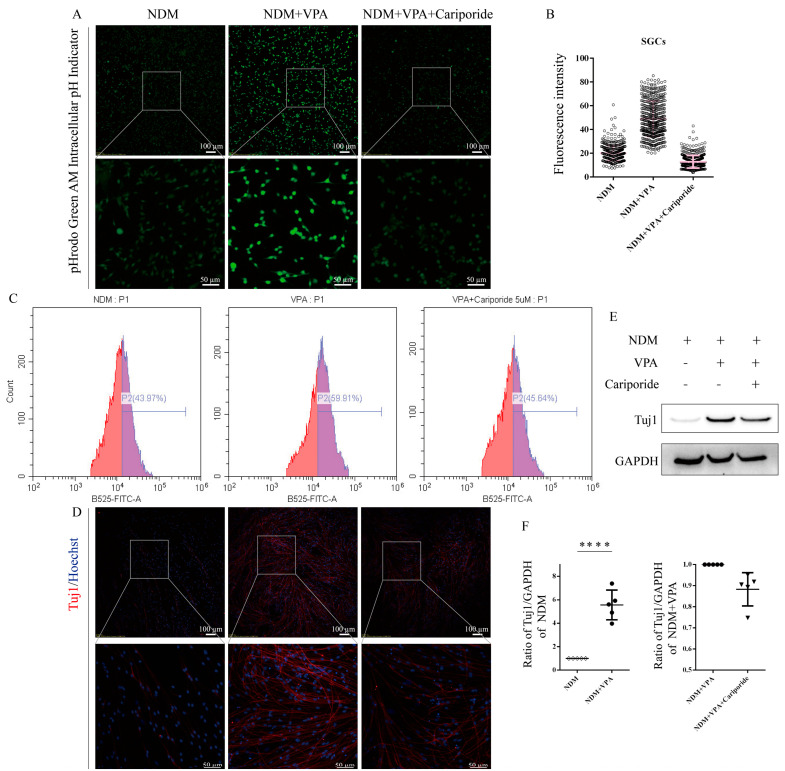
VPA promotes the neuronal differentiation of SGCs via an increase in the intracellular pH mediated by NHE1. After passage, the SGCs were divided into three groups: the NDM group, VPA group, and VPA + Cariporide group: (**A**) After 4 h, pH indicators were used to detect changes in the intracellular pH. (**B**) Fluorescence intensity statistics per cell after pH indicator treatment. The fluorescence intensity of each cell was measured from confocal microscopy images using ImageJ. At least 30 cells were counted in each image, and 4–6 images were randomly selected for statistical analysis. The data are presented as the mean ± SD of at least three independent experiments. (**C**) After incubating the intracellular pH indicator, a flow cytometer was used to detect the changes in the intracellular pH. The bar graph represents the BCECF^+^ population (P2) among all single cells (%P2), which intuitively reflects relative changes in the intracellular pH. (**D**,**E**) Representative images of immunofluorescence staining and Western blot. (**F**) Statistical analysis. The ImageJ software was used to calculate the ratio of Tuj1/GAPDH, and the ratio of Tuj1/GAPDH in the control was normalized to 1. The statistical significance of the differences was assessed using one-way analysis of variance (ANOVA), followed by Tukey’s test. The data are presented as the mean ± SD of at least three independent experiments. NDM vs. NDM + VPA: *p* < 0.0001, ****; NDM + VPA vs. NDM + VPA + Cariporide: *p* = 0.6698. Original Western blot images can be found in Figure S2.

NHE1 is a membrane protein that is ubiquitously expressed in all mammalian cells. It maintains the intracellular pH by expelling 1 intracellular proton and exchanging 1 extracellular sodium ion, thereby protecting cells from acidification [15]. We found that VPA treatment can lead to the upregulation of *NHE1* expression in SGCs, U87, U251, and SVGp12 cells (Figure 5A), suggesting that VPA may increase the intracellular pH through NHE1. To verify this hypothesis, we conducted immunofluorescence staining after VPA treatment for 3 days and found that VPA treatment did enhance the expression of NHE1 in SGCs and SVGp12 cells (Figure 5B,C). Then, we used the NHE1 inhibitor Cariporide and detected the changes in the intracellular pH via laser confocal microscopy and flow cytometry. The results showed that compared with the VPA-treated group, the fluorescence intensity of SGCs in the VPA + Cariporide-treated group was significantly weakened (Figure 6A,C), indicating impaired intracellular alkalinization. Subsequent immunofluorescence staining and WB analyses demonstrated that the addition of Cariporide attenuated the VPA-induced upregulation of the neuronal marker Tuj1 (Figure 6D,F), suggesting that NHE1 is a critical mediator of VPA-induced neuronal differentiation in SGCs.

**Figure 7:**
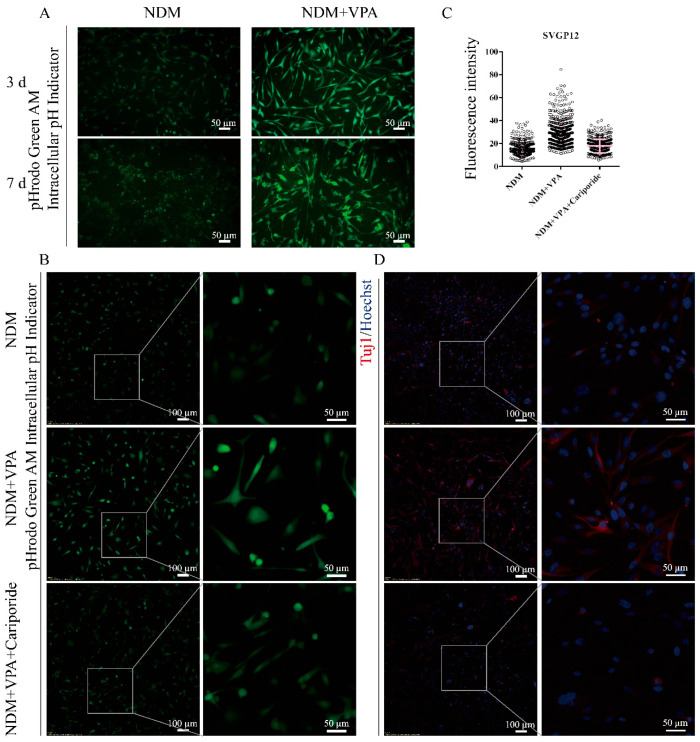
VPA modulates the intracellular pH via NHE1 to promote neuronal differentiation in SVGp12: (**A**) Representative images after incubation with the pH indicator BCECF. The SVGp12 cells were divided into two groups: the NDM group and VPA group. After treatment for 3 days and 7 days, cells were incubated with the pH indicator BCECF. (**B**) Representative fluorescence images of SVGp12 cells after 4 h of incubation with the pH indicator. (**C**) Fluorescence intensity statistics per cell after the pH indicator treatment. The fluorescence intensity of each cell was measured from confocal microscopy images using ImageJ. At least 30 cells were counted in each image, and 4–6 images were randomly selected for statistical analysis. The data are presented as the mean ± SD of at least three independent experiments. (**D**) Representative images of immunofluorescence staining. After treatment for 3 days, the cells were fixed with paraformaldehyde for immunofluorescence staining.

In addition, we used the human astrocyte cell line SVGp12 to validate our findings. The results showed that VPA treatment could also upregulate the intracellular pH of SVGp12 cells and enhance the expression of the neuron-specific marker Tuj1. After the addition of the NHE1 inhibitor Cariporide, the intracellular pH of SVGp12 cells decreased; the expression of Tuj1 was also downregulated accordingly (Figure 7). These results further prove that VPA promotes neuronal differentiation by upregulating the intracellular pH via NHE1.

### 3.4. VPA Activates β-Catenin Signaling by Increasing the Intracellular PH

**Figure 8:**
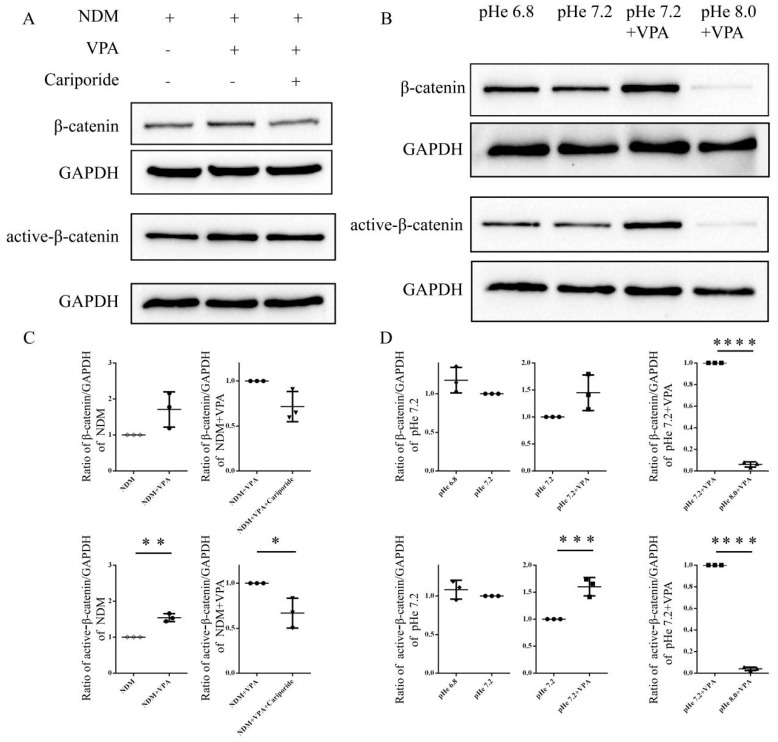
VPA activates β-catenin signaling through intracellular pH elevation: (**A**,**B**) Representative images of Western blots. (**A**) Passaged SGCs were divided into three groups: the NDM group, VPA group, and VPA + Cariporide group. This was followed by a 3-day treatment. (**B**) After passage, the SGCs were divided into four groups: the pHe 6.8 NDM group, pHe 7.2 NDM group, pHe 7.2 + VPA group, and pHe 8.0 + VPA group. They were then treated for 3 days. (**C**,**D**) Statistical analysis. The ImageJ software was used to calculate the ratio of β-catenin/GAPDH and active-β-catenin/GAPDH, and the ratio of β-catenin/GAPDH and active-β-catenin/GAPDH in the control was normalized to 1. The statistical significance of the differences was assessed using one-way analysis of variance (ANOVA), followed by Tukey’s test. The data are presented as the mean ± SD of at least three independent experiments. (**C**) β-catenin: NDM vs. NDM + VPA: *p* = 0.0644; NDM + VPA vs. NDM + VPA + Cariporide: *p* = 0.1592. ABC: NDM vs. NDM + VPA: *p* = 0.0095, **; NDM + VPA vs. NDM + VPA + Cariporide: *p* = 0.0118, *. (**D**) β-catenin: pHe 6.8 vs. pHe 7.2: *p* = 0.6606; pHe 7.2 vs. pHe 7.2 + VPA: *p* = 0.0700; pHe 7.2 + VPA vs. pHe 8.0 + VPA: *p* <0.0001, ****. ABC: pHe 6.8 vs. pHe 7.2: *p* = 0.7753; pHe 7.2 vs. pHe 7.2 + VPA: *p* = 0.0005, ***; pHe 7.2 + VPA vs. pHe 8.0 + VPA: *p* < 0.0001, ****. Original Western blot images can be found in Figure S3–S6.

Studies have shown that VPA can induce the neuronal differentiation of NSCs by activating the Wnt signaling pathway [16], which is a regulatory factor known to play a key role in neuronal differentiation. Our results further demonstrated that VPA treatment can lead to the upregulation of β-catenin expression and its activated form (ABC) in SGCs compared with the NDM group, indicating that VPA activated the β-catenin signaling pathway (Figure 8A,C). In addition, we found that, after the addition of Cariporide, the expression of β-catenin and ABC in SGCs was downregulated (Figure 8A,C), suggesting that the increase in the intracellular pH ultimately promoted the neuronal differentiation of SGCs by activating β-catenin signaling.

The results obtained by regulating the intracellular pH also further confirmed this mechanism. We found that compared with pHe 7.2, the pHe 6.8 treatment significantly increased the intracellular pH and upregulated the expression of β-catenin and ABC in SGCs (Figure 8B,D). In contrast, compared with pHe 7.2 + VPA, the pHe 8.0 + VPA treatment disrupted the upregulation of pH induced by VPA and significantly reduced the expressions of β-catenin and ABC (Figure 8B,D). The above results show that increasing the intracellular pH can activate the β-catenin signaling pathway, while decreasing the intracellular pH inhibits its activation. Together, these results show that VPA activates β-catenin signaling by upregulating the intracellular pH, thereby promoting the neuronal differentiation of SGCs.

## 4. Discussion

The HDAC inhibitor VPA is employed in the management of epilepsy and bipolar disorders. Beyond its established applications, emerging evidence has documented its therapeutic potential against febrile seizures, migraines, and cerebral neoplasms [17], alongside its neuroprotective properties [1,18,19]. These collective findings underscore VPA’s multifaceted neuromodulatory capabilities. However, clinical observations and experimental studies consistently reveal its significant teratogenicity, which manifests as congenital malformations, developmental delays, cognitive impairments, and neural tube defects [20,21]. Consequently, the systematic elucidation of VPA’s molecular mechanisms is significant for two reasons: it enables us to optimize precision pharmacotherapy for existing applications while providing a crucial foundation for the development of next-generation therapeutics with enhanced safety profiles.

Current research indicates that VPA promotes neuronal regeneration primarily by reducing the activity of HDAC [18], modulating key signaling pathways (e.g., PI3K/AKT/mTOR [22] and Wnt [16]), and upregulating neurogenic transcription factors (e.g., Ngn1, NeuroD [18], Ngn2, and Mash1 [23]), thereby enhancing the neuronal differentiation of neural stem cells (NSCs) while suppressing gliogenesis. In this study, we found that VPA can promote neuronal regeneration by upregulating the intracellular pH, revealing its neuronal regulatory mechanism and further enhancing our understanding of the mechanism via which VPA regulates neurogenesis.

Early theories posited that changes in the intracellular pH resulted in the formation of cancer or the occurrence of disease. However, recent research has shown that normal mammalian cells exhibit transient elevations in the intracellular pH during cell cycle progression, directed migration, and differentiation [8]. Using intracellular pH-sensitive indicators, we found that VPA treatment resulted in marked elevations in the intracellular pH in both SGCs and SVGp12 cells. Notably, reducing the intracellular pH significantly inhibited VPA-induced neurodifferentiation, whereas increasing the intracellular pH enhanced this process. These findings suggest that VPA-induced neurodifferentiation is closely related to the elevation of the intracellular pH.

The physiological pH gradient in normal cells is tightly regulated by multiple pH-regulatory membrane proteins, including NHE1. As the predominant isoform of the Na^+^/H^+^ exchanger family, NHE1 is ubiquitously expressed across mammalian cells. Its transmembrane domain extrudes protons by exchanging one intracellular proton for one extracellular sodium ion, thereby regulating the intracellular pH and protecting against acidotic stress [24,25,26]. Studies have demonstrated that the decrease in the intracellular pH caused by the loss or inhibition of NHE1 interferes with the neural differentiation of mouse embryonic carcinoma cells induced by retinoic acid [27]. In this study, we found that the addition of the NHE1 inhibitor Cariporide not only attenuated the elevation of the intracellular pH, but also suppressed the VPA-induced neuronal differentiation process, demonstrating that VPA regulates the intracellular pH through NHE1, thereby promoting neuronal differentiation.

We also found that an increase in the intracellular pH promoted the differentiation of SGCs into neurons, which may be closely related to the activation of β-catenin signaling. We observed that the administration of VPA significantly upregulated the expression of β-catenin in SGCs; meanwhile, the use of the NHE1 inhibitor Cariporide not only reduced the intracellular pH but also downregulated β-catenin levels. Moreover, the regulation of the intracellular pH also significantly affected the expression level of β-catenin protein. These results reveal that the elevation of the intracellular pH is a critical upstream event, mechanistically connected to VPA-induced β-catenin activation.

This aligns with established evidence demonstrating that an alkaline pH promotes the recruitment of Wnt transducer Dishevelled (Dvl) in the membrane through electrostatic interactions with Frizzled-7 (Fz) receptors, whereas an acidic pH disrupts these complexes via charge repulsion [28,29,30]. Furthermore, an elevated pH enhances β-catenin acetylation and nuclear translocation, while a reduction in pH suppresses GSK-3β phosphorylation, impairing β-catenin stabilization and nuclear entry [13,14,31]. Crucially, β-catenin signaling is crucial for neurogenesis: neural crest-specific β-catenin knockout abolishes Ngn2 expression and the development of sensory neurons [32]; meanwhile, the constitutive activation of β-catenin in NCCs promotes precocious Ngn2 expression and the overproduction of sensory neurons at the expense of other neural crest lineages [33]. Collectively, as a histone deacetylase inhibitor, VPA activates β-catenin signaling by upregulating the intracellular pH, thereby promoting neuronal differentiation. However, the precise molecular cascades that link pH dynamics to β-catenin signaling await further elucidation.

In addition, during our experiments, we observed that the intracellular pH in SGCs decreases as the extracellular pH increases, which may be related to the expression of SLC4A2/3 (AE2/AE3). Under physiological conditions, modest fluctuations in pHe are neutralized almost instantaneously by the CO_2_/HCO_3_^−^ buffer and by a “see-saw” of acid-extruding (NHE1 or NBCs) versus acid-loading (AE2/AE3, freely diffusing CO_2_) transporters; therefore, the intracellular pH usually changes in the same direction as pHe [34]. When pHe departs by ≳0.5 pH units, however, the two arms of this see-saw become unbalanced and inverse gradients appear: (1) Abrupt alkalinization raises [HCO_3_^−^]e, stimulating AE2/AE3 to export HCO_3_^−^ while importing Cl^−^/H^+^; at the same time, an alkaline pH inhibits NHE1, together producing a rapid “external-alkali/internal-acid” response known as paradoxical intracellular acidosis [35,36]. (2) Sustained acidification (e.g., in tumors or hypoxic, lactate-rich tissue) maximally activates NHE1/NBCn1 and silences AE2/AE3, yielding the opposite “external-acid/internal-alkali” gradient that characterizes cancer metabolism [37,38,39]. A similar NHE1-driven brain alkalosis precipitates seizures after neonatal asphyxia [40].

Our data show that SGCs acidify when the pHe is raised. Single-cell atlases reveal intrinsically low SLC9A1 (NHE1) but moderate-to-high AE2/AE3 expression in SGCs [41,42], pre-disposing them to the “alkali-out/acid-in” pattern. The pharmacological or epigenetic upregulation of NHE1—such as that produced by VPA—can reverse this acidification and concomitantly activate the pH-sensitive pathways (e.g., β-catenin) that promote neuro-glial differentiation [43]. Hence, whether the intracellular pH tracks or opposes pHe depends on the baseline ratio and real-time promotion of acid-extrusion versus acid-loading transporters, a principle that may be exploited to guide SGCs through the targeted modulation of pH regulators.

## 5. Conclusions

Our findings demonstrate that VPA orchestrates the neuronal differentiation of SGCs through a sequential regulatory axis: upregulating NHE1 expression, elevating the intracellular pH, and activating β-catenin signaling. This study elucidates the pH-dependent mechanism that underlies VPA-mediated neurogenesis, significantly enhancing our mechanistic understanding of HDAC inhibitor biology. Crucially, these insights provide a theoretical framework for optimizing the clinical application of VPA while proposing novel therapeutic avenues for the development of neurodifferentiation modulators with enhanced specificity and reduced off-target effects.

## Data Availability

The original contributions presented in this study are included in the article/Supplementary Materials. Further inquiries can be directed to the corresponding authors.

## Supplementary Materials

The following supporting information can be downloaded at https://www.mdpi.com/article/10.3390/biom15070986/s1, Figure S1: The original image of Figure 4B; Figure S2: The original image of Figure 6E; Figure S3: The original image of Figure 8A (β-catenin and GAPDH); Figure S4: The original image of Figure 8A (Active β-catenin and GAPDH); Figure S5: The original image of Figure 8B (β-catenin and GAPDH); Figure S6: The original image of Figure 8B (Active β-catenin and GAPDH).
