# Supplementary material for: Valproic Acid Promotes the Differentiation of Satellite Glial Cells into Neurons via the pH-Dependent Pathway
Source: Biomolecules. 2025 Jul 11;15(7):986. doi: 10.3390/biom15070986 (PMC12292801; doi:10.3390/biom15070986)

Figure S1 The original image of Figure 4B

The original image of Figure 4B (Tuj1 and GAPDH)with membrane edges visible	The original image of Figure 4B-Tuj1	The original image of Figure 4B-GAPDH

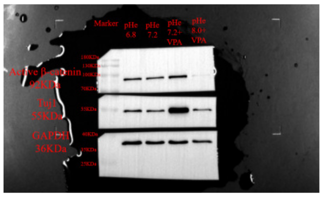

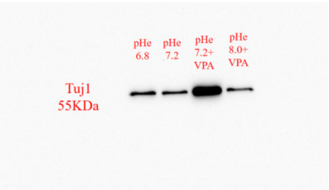

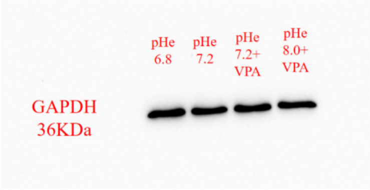

Figure S2 The original image of Figure 6E

The original image of Figure 6E (Tuj1 and GAPDH) with membrane edges visible	The original image of Figure 6E-Tuj1	The original image of Figure 6E-GAPDH

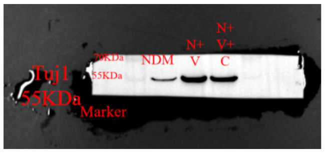

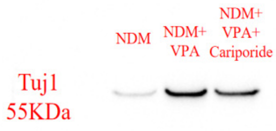

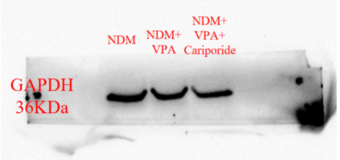

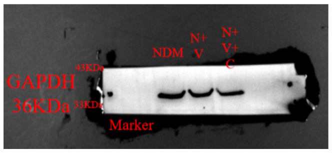

NDM: neural differentiation basic medium+PBS+DMSO

N+V: neural differentiation basic medium +VPA+DMSO

N+V+C: neural differentiation basic medium +VPA+Cariporide （NHE1 inhibitor ）

Figure S3 The original image of Figure 8A (β-catenin and GAPDH)

The original image of Figure 8A (β-catenin and GAPDH) with membrane edges visible	The original image of Figure 8A (β-catenin)	The original image of Figure 8A (GAPDH)

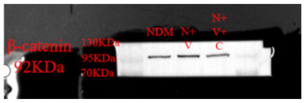

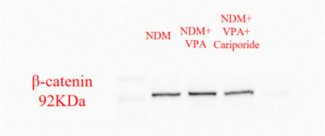

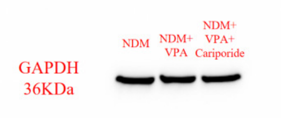

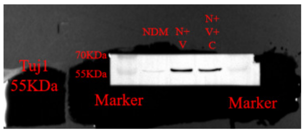

NDM: neural differentiation basic medium+PBS+DMSO

N+V: neural differentiation basic medium +VPA+DMSO

N+V+C: neural differentiation basic medium +VPA+Cariporide （NHE1 inhibitor ）

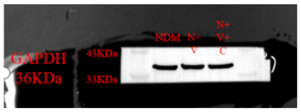

Figure S4 The original image of Figure 8A (Active β-catenin and GAPDH)

The original image of Figure 8A (Active β-catenin and GAPDH) with membrane edges visible	The original image of Figure 8A (Active β-catenin)	The original image of Figure 8A (GAPDH)

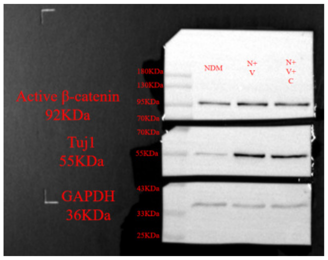

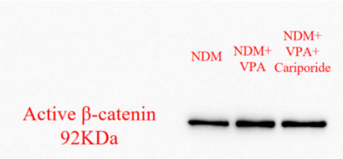

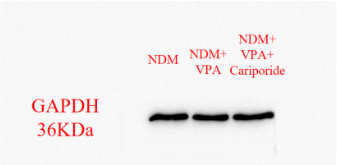

Figure S5 The original image of Figure 8B (β-catenin and GAPDH)

The original image of Figure 8B (β-catenin and GAPDH) with membrane edges visible	The original image of Figure 8B (β-catenin)	The original image of Figure 8B (GAPDH)

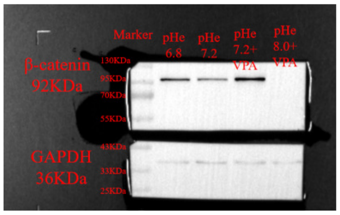

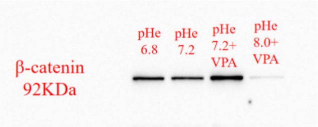

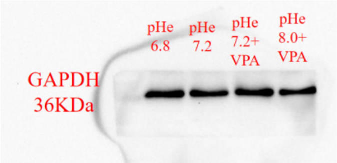

Figure S6 The original image of Figure 8B (Active β-catenin and GAPDH)

The original image of Figure 8B (Active β-catenin and GAPDH) with membrane	The original image of Figure 8B (Active β-catenin)	The original image of Figure 8B (GAPDH)

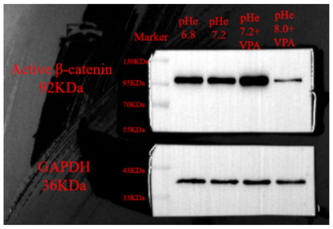

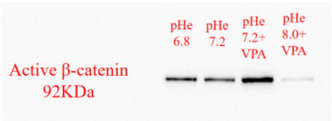

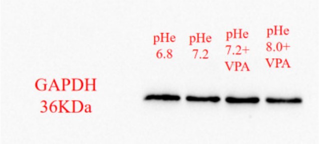

Supplement: Supplementary file 1 [file biomolecules-15-00986-s001.zip › biomolecules-3668361-supplementary.pdf]
